# Synergistic and Pharmacotherapeutic Effects of Gemcitabine and Cisplatin Combined Administration on Biliary Tract Cancer Cell Lines

**DOI:** 10.3390/cells8091026

**Published:** 2019-09-03

**Authors:** Yasunari Sakamoto, Seri Yamagishi, Takuji Okusaka, Hidenori Ojima

**Affiliations:** 1 Department of Hepatobiliary and Pancreatic Oncology, National Cancer Center Hospital, Tokyo 104-0045, Japan (Y.S.) (T.O.); 2 Department of Gastroenterology and Hepatology, International University of Health and Welfare Atami Hospital, Shizuoka 413-0012, Japan; 3 Division of Cancer Genomics, National Cancer Center Research Institute, Tokyo 104-0045, Japan; 4 Department of Pathology, Keio University School of Medicine, Tokyo 160-0016, Japan

**Keywords:** gemcitabine, cisplatin, combined administration, synergistic effect, pharmacotherapeutic effect, biliary tract cancer

## Abstract

Gemcitabine (GEM) and cisplatin (CDDP) combination therapy (GC) is the standard chemotherapy for advanced biliary tract cancer (BTC); however, its pharmacotherapeutic efficacy remains unclear. To investigate the effects of GC, we selected 11 from 17 BTC cell lines, according to their GEM sensitivity, to be assessed using the MTS assay. The presence of synergistic effects of GC was determined using the Bliss additivism model (BM) and the combination index (CI) at a GEM:CDDP molar ratio of 7:1; this ratio was based on the respective human renal clearances of the two drugs. The pharmacotherapeutic effects were evaluated by comparing the IC50 values for administrations of GEM alone and GC in combination. All cell lines showed synergistic effects when analyzed using the BM. Based on the CI values, strong synergism, synergism, and additive effects were seen in four, five, and two cell lines, respectively. For all four GEM-resistant cell lines, on which GC had strong synergistic effects, the pharmacotherapeutic effects of GC were disappointing, with all IC50 values > 1 µM. For the GEM-effective cell lines, on which GC had synergistic or additive effects, the IC50 values were all <1 µM, and the differences were small between the IC50s for administration of GEM alone and GC in combination. Our results suggest that GC has synergistic effects on BTC cell lines but that its pharmacotherapeutic effects are inadequate.

## 1. Introduction

Biliary tract cancer (BTC) is a highly malignant disease for which surgical resection is the only curative therapy. Moreover, there is no effective chemotherapy for recurrent or inoperable cases. Consequently, the development of new therapeutic anticancer drugs for BTC is eagerly anticipated. Recently, several studies on potential new therapeutic molecular targets for BTC, including tumors with genomic alterations, were described [1,2,3,4,5,6]. Since 2010, gemcitabine (GEM) and cisplatin (cis-diamminedichloroplatinum, CDDP) combination therapy (GC) has been shown to significantly extend the median overall survival in BTC patients compared with GEM monotherapy [7]. Currently, GC therapy is the only standard chemotherapy regimen accepted worldwide. However, for some patients the clinical efficacy of GC is not satisfactory.

The synergistic mechanism of combined GC therapy is considered to be the ability of GEM to become incorporated into DNA, thereby promoting an increase in the accumulation of platinum compounds, such as CDDP, and the formation of CDDP–DNA adducts. Subsequently, GEM is converted to 2’-deoxy-5-azacytidine, and the CDDP–DNA adducts reduce cells’ ability to repair DNA [8,9]. However, the specific mechanism of the synergistic effect and the true therapeutic impact of combined GC administration remain unclear. To understand the synergistic effects of GC therapy and to improve outcomes for patients with advanced or recurrent BTC, we consider that the efficacy of GC should first be elucidated in a preclinical-style study. The synergistic effects of GC administration in vitro have been reported for ovarian cancer, head and neck cancer, lung cancer, neuroblastoma, and bladder carcinoma [8,9,10,11,12,13,14]. However, as far as we know, there have been no detailed studies on the pharmacotherapeutic activity, synergistic effect, and plasma clearance of GC. In our previous work, we established a number of BTC cell lines and performed in vitro sensitivity studies for several anticancer drugs, including GEM [15,16]. Some of our BTC cell lines showed resistance to GEM. Therefore, these BTC-related bioresources can be used to examine in detail the validity and pharmacotherapeutic activity of GC in highly practical preclinical-style studies.

The aim of this study was to clarify both the synergistic effect of CDDP administration in combination with GEM and its real pharmacotherapeutic activity on BTC cell lines and to demonstrate the basic premise of the efficacy of GC in the clinical setting.

## 2. Materials and Methods

### 2.1. Cell Culture

In the current study, we used 17 BTC cell lines: seven extrahepatic BTC cell lines, nine intrahepatic BTC cell lines, and one gallbladder carcinoma cell line. Of these cell lines, 13 were our established cell lines: five were derived from extrahepatic BTC (NCC-BD1, NCC-BD2, NCC-BD3, NCC-BD4-1, and NCC-BD4-2) and eight were derived from intrahepatic BTC (NCC-CC1, NCC-CC3-1, NCC-CC3-2, NCC-CC4-1, NCC-CC4-2, NCC-CC4-3 (NCC-CC5), NCC-CC6-1, and NCC-CC6-2). Further information regarding these cell lines is presented in Table 1 [15,16]. Of the other four cell lines, three (HuCCT1, TKKK, and TGBC24TKB) were obtained from Riken Bio Resource Center (Tsukuba, Japan) and one (OZ) was obtained from the Japanese Collection of Research Bioresources (Osaka, Japan). The HuCCT1 and OZ cell lines were derived from extrahepatic cholangiocarcinoma, the TKKK cell line was derived from intrahepatic bile duct carcinoma, and the TGBC24TKB cell line was derived from gallbladder carcinoma. Our 13 established BTC cell lines and HuCCT1 were cultured in RPMI-1640 medium (Sigma-Aldrich; St. Louis, MO, USA), whereas OZ, TKKK, and TGBC-24TKB were cultured in Dulbecco’s Modified Eagle’s Medium (Sigma-Aldrich). In both media, fetal bovine serum (Thermo Fisher Scientific, Waltham, MA, USA) and penicillin–streptomycin–glutamine (Thermo Fisher Scientific) were added to make concentrations of 10% and 1%, respectively. All cell lines were cultured in an incubator at 37 °C at 5% CO_2_.

### 2.2. Chemicals and Reagents

GEM (gemcitabine hydrochloride), which was obtained from Eli Lilly Japan K.K. (Kobe, Japan), was dissolved in Dulbecco’s phosphate buffered saline (Sigma-Aldrich). Cisplatin (Wako-Junyaku, Osaka, Japan) was dissolved in dimethyl sulfoxide (DMSO; Sigma-Aldrich). Solutions of cisplatin in DMSO were freshly prepared before use. The concentration of DMSO in medium was adjusted so as not to exceed 0.2%, which was ascertained as the concentration that had no influence on any cell line.

### 2.3. Chemosensitivity Examination

#### 2.3.1. Single-Agent Examination and Stratification

The effects of GEM and/or CDDP on 17 BTC cell lines were evaluated using the 3-(4,5-dimethylthiazol-2-yl)-5-(3-carboxymethoxyphenyl)-2-(4-sulfophenyl)-2H-tetrazolium (MTS) cell viability assay. Cells were placed in each well of a 96-well plate at 3.0 × 10^3^/ well in 100 μL culture medium. After 24 h, the medium was removed and replaced with GEM and/or CDDP at concentrations in the range 0–100 µM. The data for GEM administration for our 13 established cell lines were obtained from our previous report [15,16]. For HuCCT1, TKKK, OZ, and TGBC24TKB, the same concentrations in the range 0–100 μM (0, 0.0001, 0.00033, 0.00067, 0.001, 0.0033, 0.0067, 0.01, 0.033, 0.067, 0.1, 0.33, 0.67, 1, 3.3, 6.7, 10, 33, 67, and 100 μM) as those in our previous reports were used [15,16]. The CDDP concentrations for addition to all cell lines were also set in the range 0–100 μM (0, 0.001, 0.01, 0.1, 1, 10, 20, 30, 50, 75, and 100 μM). These concentrations were considered appropriate with reference to previous reports regarding the IC50 values of CDDP [9,12]. After 72 h of drug administration, we used CellTiter 96 (Promega Corporation, Madison, WI, USA) and then measured the absorbance by the MTS assay using the AQueous One Solution Cell Proliferation Assay (Promega Corporation, Madison, WI, USA) according to the manufacturer’s protocol. To ensure the reproducibility of cell viability data after administration of GEM alone and CDDP alone, we seeded cell lines to nine and six wells, respectively, for each concentration. The data were averaged for each concentration. To confirm that the condition of the cell lines was acceptable, and to allow data from previous studies to be validly imported into the current study, we compared the cell proliferation data after GEM single administration with the equivalent data recorded in earlier studies and found them to be consistent.

For GEM, the cell viability curves and the 50%, 60%, 70%, and 80% inhibitory concentrations (IC50–80) were then calculated for each cell line; for CDDP, the cell viability curves and the 50% inhibitory concentrations (IC50) were calculated for a subset of cell lines (Table 1 and Table 2). The BTC cell lines were stratified according to their GEM sensitivity into three groups: effective (IC50 < 1 µM and IC70 was calculable), resistant (IC50 could not be determined), and intermediate (IC60 and/or IC50 could be determined, but IC70 could not be determined). For CDDP, cell lines with IC50 ≤ 50 µM were stratified as effective because we assessed that drug toxicity was superior to drug efficacy at high concentrations of CDDP [17].

#### 2.3.2. Analysis of the Efficacy of GC Using the Bliss Additivism Model

We evaluated the efficacy of GC using the Bliss additivism model (BM). The experimental method was adjusted from that used for the single administrations of GEM and CDDP: following the placement of cells into 96-well plates, an amount of medium similar to that administered in the single-agent experiments was removed from each well. Consequently, 50 μL of each drug was prepared at double concentrations to make up 100 μL of GC. The GEM concentrations were set in the range 0–10 μM (0, 0.001, 0.01, 0.1, 1, and 10), and the CDDP concentrations were set in the range 0–50 μM (0, 0.01, 0.1, 1, 10, and 50). Each combination of concentrations of GEM and CDDP were tested. After 72 h of drug administration, we measured absorbances using the MTS assay in a manner similar to that for single-agent administration. The degree of efficacy of GC was determined using the BM [18,19,20,21] by applying the values obtained from the MTS assay. To ensure the reproducibility of the GC cell viability data used for BM analysis after administration of GEM and CDDP in combination, we seeded cell lines to three wells for each combination of concentrations. These three values were then averaged. To assess the condition of the cell line, we compared the cell proliferation data after the administration of GEM alone with the equivalent data recorded in earlier studies and found them to be acceptable.

The BM was calculated for each combination of concentration of drugs A and B using the following expression: Ebliss = EA + EB − (EA × EB). EA and EB are the fraction depression effects caused by drugs A and B separately at each concentration (fractional inhibitory effects), and Ebliss is the inhibition ratio expected if the two drugs exhibited only an additive effect. The BM value is the difference between the observed cell viability data and that expected if the effects of combined administration are purely additive (the latter being the Ebliss value). A BM of 0 indicates additive effects, BM < 0 indicates antagonistic effects, and BM > 0 indicates synergistic effects. To evaluate the effects of GC over the whole concentration range, we carried out experiments for all 36 concentration combinations for each cell line. However, to avoid the influence on the apparent drug efficacy of drug toxicity in the high concentration range of CDDP, we focused on data near the concentration range for which the IC50 of CDDP was calculable. These concentration ranges for each cell line are those inside the black lines in in the BM matrices. 

#### 2.3.3. Analysis of the Efficacy of GEM–CDDP at a Molar Ratio of 7:1 

##### Combination Study

For advanced BTC, based on the drug package inserts, the clinical dose of GC treatment is GEM 1000 mg/m^2^ and CDDP 25 mg/m^2^, giving a dose ratio of 40:1. The human plasma clearances of GEM and CDDP are 85.6 and 15 L/h/m^2^, respectively, giving a plasma concentration ratio for GEM:CDDP of 5.6:1 [22,23,24]. Therefore, we examined the efficacy of GC treatment using an adjusted dose of GEM:CDDP giving a molar ratio of approximately 7:1, which was close to the clinical plasma ratio for CDDP administration in combination with GEM. After cells had been conditioned in wells for 24 h, we removed an amount of medium similar to that for single-agent administration and replaced it with GEM and CDDP at a molar ratio of 7:1 based on GEM concentrations in the range 0–100 μM (0, 0.001, 0.01, 0.05, 0.1, 0.5, 1, 5, 10, and 100 μM). At 72 h after drug administration, we conducted absorbance measurement for the MTS assay. To ensure the reproducibility of the cell viability data that were used for combination index (CI) analysis after administration of GC at a molar ratio of 7:1, nine wells were used for each concentration of GC. The data for each concentration were then averaged. To assess the condition of the cell lines, we also obtained cell proliferation data for the administration of GEM alone using three wells for each concentration. The results indicated that the cell lines were in an acceptable condition. To calculate the CI, data are required for the effects of administration of CDDP alone. Consequently, we used single-administration CDDP data at concentrations of 0, 0.001, 0.01, 0.1, 1, 10, 20, 30, 50, and 100 μM. We evaluated the efficacy of GC administration by using the CI [25], which was calculated using CalcuSyn software (Biosoft, Cambridge, UK). For the CI calculation, growth inhibition values (fraction affected, Fa) were used. There are several definitions of CI, and we used that given in “Drug combination studies and their synergy quantification using the Chou–Talalay method” [25]. The average CI was determined only when Fa was greater than or equal to 0.2. We considered that CI ≤ 0.3 indicated strong synergism, 0.3 < CI ≤ 0.7 indicated synergism, 0.7 < CI ≤ 1.25 indicated an additive effect only, and 1.25 < CI indicated antagonism [26] (Table 2). After calculation, we constructed dose–response curves for GC and for GEM alone for each cell line and stratified the cell lines into three groups based on their sensitivity to GEM. We then evaluated the variability among the cell line groups of the differences in drug effects between GC at a molar ratio of 7:1 and GEM single administration using the mean data from multiple experiments at each concentration.

##### Statistical Analysis

Student’s *t*-test was performed using Microsoft Excel 2016 for Windows (Microsoft Corporation; Redmond, WA, USA) to test the statistical significance of the differences between the dose–response curves of GC and GEM at each concentration for all cell lines. Statistical significance was set at *P* < 0.05.

### 2.4. Ethics

This study was approved by the ethics committee of the National Cancer Center (ID: 2007-022).

## 3. Results

### 3.1. Evaluation of GEM and CDDP Single-Agent Administration

**Figure 1:**
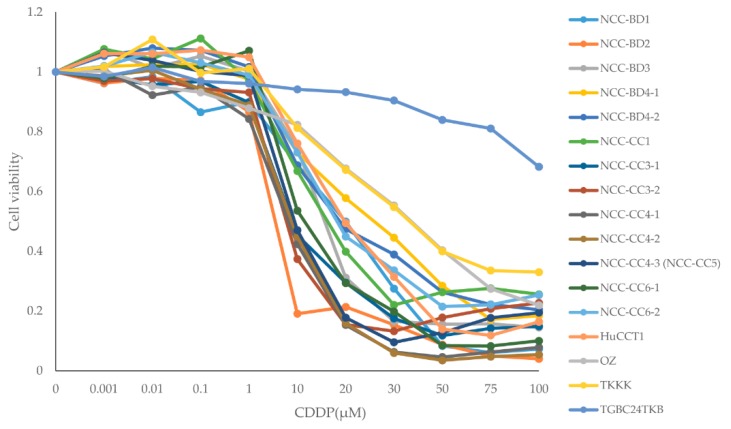
Dose–response curves of biliary tract carcinoma cell lines for cisplatin (CDDP) single administration. The dose–response curves for each cell line were used to calculate IC50 values.

The dose–response analysis for single administrations of GEM and CDDP are given for all BTC cell lines in Table 1 and Figure 1 and the IC50 values of CDDP are shown in Table 2. Based on the IC50 values, GEM single administration was considered to be effective for cell lines NCC-BD4-1, BD4-2, CC3-1, CC3-2, NCC-CC4-3 (NCC-CC5), CC6-1, HuCCT1, and TGBC24TKB. Cell lines NCC-BD2, BD3, OZ, and TKKK were resistant to GEM, and cell lines NCC-BD1, CC1, CC4-1, CC4-2, and CC6-2 showed intermediate effects. CDDP was effective for 16 cell lines (94%), with IC50 values of 3.49 to 35.94 µM, but TGBC24TKB was resistant to CDDP (Figure 1 and Table 2). The cell line TGBC24TKB was resistant to CDDP, but GEM was effective against it. In contrast, cell lines NCC-BD2 and NCC-BD3 were resistant to GEM, but CDDP was effective against them. Based on these results, we selected 11 representative BTC cell lines for further study: NCC-BD4-2, CC6-1, HuCCT1, and TGBC24TKB as lines for which GEM is effective; NCC-BD2, NCC-BD3, OZ, and TKKK as GEM resistant; and NCC-BD1, CC1, and CC4-1 as intermediate.

### 3.2. Evaluation of GC Combination Using the Bliss Additivism Model

**Figure 2:**
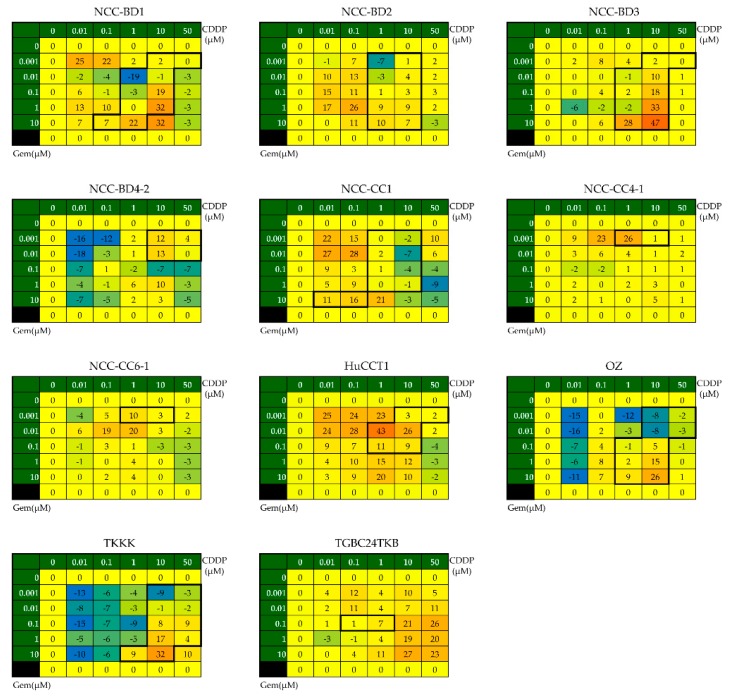
Evaluation of the combined effects of GC administration using the Bliss additivism model (BM). We determined the BM matrices to evaluate synergistic effects, focusing on the concentration ranges for which IC50 for CDDP was calculable. These concentration ranges are enclosed in the black frames on the heat maps of the BM matrices for each cell line. The mean value of the sum of the data for each cell in the matrices was calculated; a value of >1 was designated as BM positive (i.e., there was a synergistic effect).

The results of BM analysis for each cell line are shown in Figure 2. As mentioned in Section 2.4, we evaluated the synergy for each cell line using the BM matrix by focusing on the concentration ranges for which the IC50 for CDDP was calculable (data within the black frames). GC had synergistic effects against all 11 cell lines, including four GEM-resistant cell lines (Figure 2, Table 2).

### 3.3. Synergy or Additivism for GEM:CDDP Combination at a Molar Ratio of 7:1

**Figure 3:**
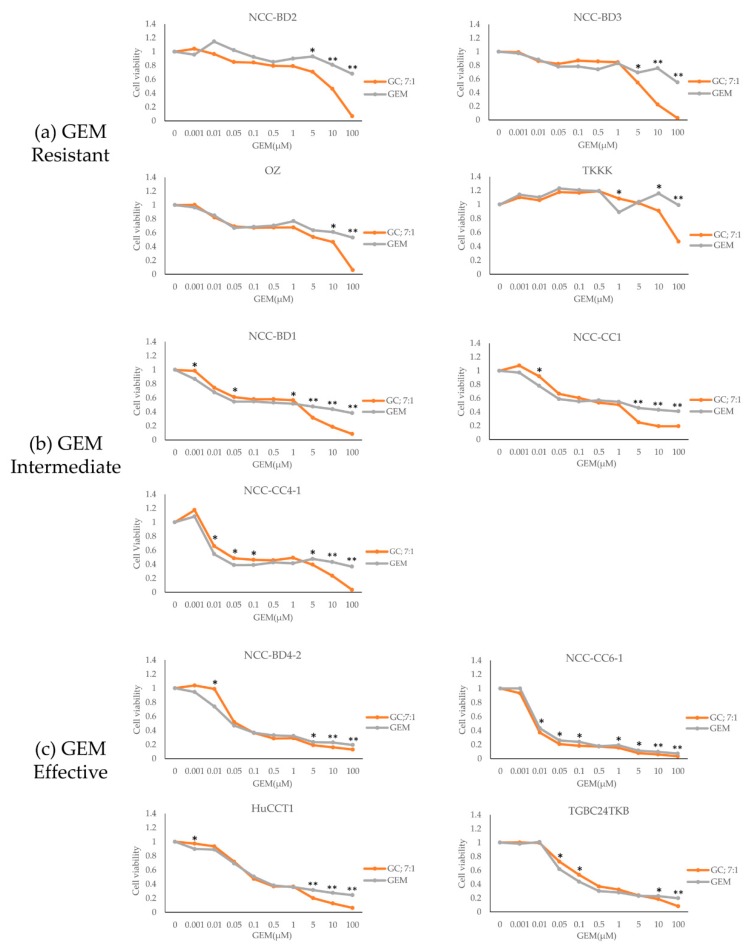
The dose–response curves for combined administration of GEM–CDDP at a molar ratio of 7:1 and administration of GEM alone. Cell lines are stratified into three groups based on their sensitivity to GEM: (**a**) GEM resistant group, (**b**) GEM intermediate group, and (**c**) GEM effective group. There was a difference between the IC50 levels for GC and GEM alone for the four GEM-resistant cell lines. The dose–response curves for GC and GEM alone were compared. * *P* < 0.05 and ** *P* < 0.0001 by Student’s *t*-test. Statistical significance was set at *P* < 0.05.

The dose–response curves for combined administration of GEM and CDDP at a molar ratio of 7:1 and for GEM single administration against all BTC cell lines are shown in Figure 3; the graphs are stratified based on the cell lines’ sensitivity to GEM alone. Strong synergism was seen in four cell lines (36%; NCC-BD2, BD3, OZ, and TKKK) that were all GEM resistant. Synergism was seen in five cell lines (45%; NCC-BD4-2, CC1, CC4-1, HuCCT1, and TGBC24TKB) and additive effects were seen in two cell lines (18%; NCC-BD1 and CC6-1). For no cell line was there an antagonistic effect of combined GEM and CDDP (Table 2). 

### 3.4. The True Pharmacotherapeutic Effect of GC Compared with That of GEM Single Administration

To evaluate the true pharmacotherapeutic effects of GC, we compared the IC50 values between GEM single administration and GC at a molar ratio of 7:1. All four GEM-resistant cell lines showed measurable IC50 values for GC at a 7:1 molar ratio, but all four IC50 values were >1 µM. In contrast, all four GEM-effective cell lines had measurable IC50 values for GC at a 7:1 molar ratio, but all four IC50 values were <1 µM. In the three GEM-intermediate cell lines, GC at 7:1 molar ratio showed either synergistic or additive effects, with IC50 values in the range 0.04–1.53 (Table 2).

## 4. Discussion

In the present study, we used 11 representative BTC cell lines and multiple methods to measure drug efficacy, allowing us to examine the relationship between the synergistic effects and pharmacotherapeutic activity of combined GC administration. Our results showed a synergistic effect of GC administration in most of the BTC cell lines, even for GEM-resistant cell lines. These results suggested that the addition of CDDP to GEM represents an effective and valuable therapy for BTC. However, interestingly, although GC at a molar ratio of 7:1 showed strong synergism in all four GEM-resistant cell lines, the IC50 values of GC were relatively high (>1 µM). In addition, the average IC50 value for GEM alone was low (0.06 µM) for the GEM-effective cell lines in our study; studies on other types of cancer cell lines have found IC50s for GEM in the range 0.0025–50 µM [9,12,27,28]. The IC50 values of the four GEM-resistant cell lines that showed synergism by GC combination administration were higher than those of the GEM-effective cell lines. Therefore, we believe that the pharmacotherapeutic effects of GC at a 7:1 molar ratio were insufficient in GEM-resistant cells. Moreover, in GEM-effective cell lines, only slight differences were observed in the dose–response curves or IC50 values of GEM single administration and GC at a 7:1 molar ratio. Therefore, the presence of synergistic effects may not always mean effective pharmacotherapeutic activity, and pharmacotherapeutic activity should not be evaluated by the presence of synergistic effects alone.

Although synergistic effects of GC for other cancer cell lines have been reported [9,11,12,14], the methodologies, drug concentrations, and other quantitations were different among the reports. In the current study, we examined the synergistic effects of GEM and CDDP using two assessment methods, BM and CI. BM has merit in terms of its ability to evaluate the synergistic effects of all the combinations of concentrations of each drug without calculating IC50 values [18,19,20,21]. We selected the IC50 value range from the CDDP-based GEM concentration range while avoiding the high CDDP concentration range because we considered that the cells would likely be influenced by the high-grade cell toxicity of CDDP within the high concentration range. GC evaluation based on BM indicated synergistic effects in all cell lines, and evaluation of GC at a 7:1 molar ratio based on the CI revealed that all cell lines exhibited either synergistic or additive effects. One possible reason for this difference could be that the effects were not necessarily associated with the drug concentration [14,29]. These facts suggested the possibility of complex pharmacological factors in drug combinations. Therefore, the differences in the synergistic or additive effects probably depend on the measurement or calculation methodologies used. Nevertheless, our results showed synergistic or additive effects for combined GC administration. Furthermore, the locations of the tumors on which our established cell lines were based were classified as extrahepatic (including distal BD and hilar BD) or intrahepatic. The histologic types of the original cell line tumors were all moderately differentiated adenocarcinoma (Table 1). There were no definite correlations between the locations of the original tumors and the sensitivity of the cell lines to GEM or CDDP. Therefore, we suggest that the differences in drug sensitivities between cell lines may also depend on other factors, e.g., gene expression, drug metabolism, and biological behavior. We consider that further detailed study is needed. 

In the current study, most of the BTC cell lines showed synergistic effects of GC compared with GEM single administration. However, in the ABC-02 study of 410 patients with locally advanced or metastatic cholangiocarcinoma, gallbladder cancer, or ampullary cancer, the clinical response rates were 26.1% for GC therapy and 15.5% for GEM monotherapy [7]. As shown in our results, although GC at 7:1 showed strong synergism in GEM-resistant cell lines compared with GEM single administration, and the IC50 values all became measurable, the effects of GC administration were seen only with high drug concentrations. Furthermore, in the cell lines that showed little synergistic effect (i.e., GEM intermediate and effective cell lines), the differences were small between the IC50s for administration of GEM alone and GC in combination. According to our statistical analysis for each concentration for all cell lines, at concentrations close the IC50 value of GC, no significant differences of drug effects between GC and GEM alone were evident in two of the four GEM-effective cell lines (Figure 3). This implied that even if GC administration had synergistic effects, its pharmacotherapeutic effects were not consistent. These results suggested one of the pharmacological reasons for the inadequate clinical efficacy of GC. Based on our results, enhancing the therapeutic performance of GC in resistant cases would require the addition of a third therapeutic agent to the GC combination. A combination of GEM, CDDP, and S-1, which is an oral 5-fluorouracil derivative, (GCS) was demonstrated to have survival benefits over GC for advanced BTC in the KHBO1401-MITSUBA trial [30]. Moreover, if the discrepancy between the synergistic and pharmacotherapeutic effects of GC can be further clarified, selection of GEM-resistant cases could have important implications. Ultimately, the active use of more highly sensitive GEM efficacy markers, such as hENT1 [31], RRM1 [32], and MAGEH1 [16], as diagnostic adjuncts may improve the outcomes of patients with BTC. 

To account for the differences between in vitro and in vivo drug metabolism, we adopted a GC molar ratio of 7:1, taking into consideration the human plasma clearances of GEM and CDDP. However, in the clinical setting, liver and/or kidney drug metabolism may vary because of individual differences. Moreover, we carried out several repeat experiments with the same cell lines, and the dose–response curves were almost identical, although the IC50 values sometimes varied a little. However, the IC50 for NCC-CC1 was different between our in house data for GEM and for GEM single administration carried out as part of the combination study, despite the two dose–response curves being similar in shape and position. Therefore, further detailed studies, such as in vivo studies using xenograft models, are necessary. Such investigations may show synergistic effects of GEM and CDDP under conditions that more closely resemble those in humans.

In conclusion, the synergistic effects of GC were examined in vitro in this preliminary report using 11 BTC cell lines and two different methodologies. In one approach, we considered the GEM and CDDP plasma clearances and used a molar ratio of 7:1 to reproduce the respective concentrations in the clinical setting as much as possible. GC had synergistic or additive effects for all cell lines. However, in some cell lines for which GEM single administration was effective, the pharmacotherapeutic efficacy of GC did not show much improvement. Furthermore, in cell lines for which GEM single administration was ineffective, GC showed strong synergy but insufficient pharmacotherapeutic activity. We believe that these preclinical-style findings are important to help improve the chemotherapeutic treatment strategy for advanced and recurrent BTC.

## Figures and Tables

**Table 1:** IC50–IC80 values for GEM administration for each BTC cell line.

Cell Line	Pathological Diagnosis of Original Tumor	Location of Original Tumor	Histologic Type of Original Tumor	GEM Sensitivity	IC50 (µM)	IC60 (µM)	IC70 (µM)	IC80 (µM)
NCC-BD1 *	EHCC	Distal BD	Adeno, mod ^†^	Int	7.66	58.00	N/A	N/A
NCC-BD2 *	EHCC	Distal BD	Adeno, mod	Res	N/A	N/A	N/A	N/A
NCC-BD3 *	EHCC	Distal BD	Adeno, mod	Res	N/A	N/A	N/A	N/A
NCC-BD4-1 *	EHCC	Hilar BD	Adeno, mod	Eff	0.04	0.06	0.09	2.93
NCC-BD4-2 *	EHCC	Hilar BD	Adeno, mod	Eff	0.06	0.07	0.19	5.37
NCC-CC1 *	IHCC	Intrahepatic	Adeno, mod	Int	86.78	N/A	N/A	N/A
NCC-CC3-1 *	IHCC	Intrahepatic	Adeno, mod	Eff	0.04	1.82	9.31	85.21
NCC-CC3-2 *	IHCC	Intrahepatic	Adeno, mod	Eff	0.10	1.92	43.83	N/A
NCC-CC4-1 *	IHCC	Intrahepatic	Adeno, mod	Int	0.05	4.08	N/A	N/A
NCC-CC4-2 *	IHCC	Intrahepatic	Adeno, mod	Int	0.03	11.53	N/A	N/A
NCC-CC4-3 (NCC-CC5) *	IHCC	Intrahepatic	Adeno, mod	Eff	0.06	4.92	95.10	N/A
NCC-CC6-1 *	IHCC	Intrahepatic	Adeno, mod	Eff	0.01	0.02	0.06	3.76
NCC-CC6-2 *	IHCC	Intrahepatic	Adeno, mod	Int	10.98	35.67	N/A	N/A
HuCCT1	EHCC	N/A	N/A	Eff	0.09	0.25	2.16	8.13
OZ	EHCC	N/A	N/A	Res	N/A	N/A	N/A	N/A
TKKK	IHCC	Intrahepatic	N/A	Res	N/A	N/A	N/A	N/A
TGBC24TKB	GB Ca	GB	N/A	Eff	0.05	0.07	1.23	N/A

* Data from a previous report [15]; ^†^ moderately differentiated adenocarcinoma; IC50, 50% inhibitory concentration; IC60, 60% inhibitory concentration; IC70, 70% inhibitory concentration; IC80, 80% inhibitory concentration; EHCC, extrahepatic cholangiocellular carcinoma; IHCC, intrahepatic cholangiocellular carcinoma; GB Ca, gallbladder carcinoma; BD, bile duct; GB, gallbladder; N/A, not available, i.e., could not be determined; Eff, effective; Res, resistant; Int, intermediate.

**Table 2:** IC50 values and the effects of combination GC chemotherapy based on the Bliss index and the combination index.

Cell Line	GEM Sensitivity	GEM Single	CDDP Single	GEM:CDDP Combination				
				GEM:CDDP; 7:1 molar ratio			Bliss additivism model	
		IC50 (µM)	IC50 (µM)	IC50 (µM)	CI value	Decision	BM	Decision
NCC-BD1	Int	18.62	19.94	1.53	1.03	+/−	59.17	+
NCC-BD2	Res	N/A	3.49	8.97	0.24	2+	34.40	+
NCC-BD3	Res	N/A	14.78	5.53	0.13	2+	136.18	+
NCC-BD4-2	Eff	0.04	18.39	0.05	0.38	+	11.85	+
NCC-CC1	Int	2.38	15.41	1.04	0.58	+	13.80	+
NCC-CC4-1	Int	0.02	6.58	0.04	0.46	+	27.23	+
NCC-CC6-1	Eff	0.01	11.07	0.01	0.71	+/−	12.42	+
HuCCT1	Eff	0.11	19.68	0.09	0.48	+	93.17	+
OZ	Res	N/A	35.94	7.21	0.20	2+	36.27	+
TKKK	Res	N/A	35.34	84.66	0.09	2+	64.60	+
TGBC24TKB	Eff	0.08	N/A	0.14	0.70	+	8.40	+

IC50, 50% inhibitory concentration; CI, combination index; BM, Bliss additivism model; N/A, could not be determined; Eff, effective; Res, resistant; Int, intermediate; 2+, strong synergism; +, synergism; +/−, additive.
